# Recent advances in the technology of anesthesia

**DOI:** 10.12688/f1000research.24059.1

**Published:** 2020-05-18

**Authors:** Christian Seger, Maxime Cannesson

**Affiliations:** 1 Department of Anesthesiology and Perioperative Medicine,UCLA David Geffen School of Medicine, University of California, 757 Westwood Plaza, Los Angeles, CA, 90095, USA

**Keywords:** Artificial intelligence, technology, closed loop

## Abstract

The practice of anesthesiology is inextricably dependent upon technology. Anesthetics were first made possible, then increasingly safe, and now more scalable and efficient in part due to advances in monitoring and delivery technology. Herein, we discuss salient advances of the last three years in the technology of anesthesiology.

Consumer technology and telemedicine have exploded onto the scene of outpatient medicine, and perioperative management is no exception. Preoperative evaluations have been done via teleconference, and copious consumer-generated health data is available. Regulators have acknowledged the vast potential found in the transfer of consumer technology to medical practice, but issues of privacy, data ownership/security, and validity remain.

Inside the operating suite, monitoring has become less invasive, and clinical decision support systems are common. These technologies are susceptible to the “garbage in, garbage out” conundrum plaguing artificial intelligence, but they will improve as network latency decreases. Automation looms large in the future of anesthesiology as closed-loop anesthesia delivery systems are being tested in combination (moving toward a comprehensive system).

Moving forward, consumer health companies will search for applications of their technology, and loosely regulated health markets will see earlier adoption of next-generation technology. Innovations coming to anesthesia will need to account for human factors as the anesthesia provider is increasingly considered a component of the patient care apparatus.

## Introduction

The modern practice of anesthesiology is inextricably dependent upon technology. This dependence is not as strong among the other medical specialties and makes a review of recent advances particularly germane to the determination of our field’s future. Technology first made anesthesia possible, then safe, and will seek to make it increasingly scalable and efficient in health-care systems pressed for resources both economic and human ^1^. Herein, we discuss salient advances occurring over the last three years, focusing on automation, monitoring, and decision support systems. We lastly begin a discussion of innovation landscape in anesthesiology during the 21st century.

## Automation in the delivery of anesthetics

Equipment used to perform any task, even complicated tasks like providing an anesthetic, can be described along a spectrum from tooling to automation. A tool is powered directly by its user, whereas a machine augments its user’s input via some external power source but remains directly under user control. The hallmark of automation is the ability of a machine to alter its function without direct user input but in pursuit of a user-defined objective. Anesthesiologists used mostly tools and machines at the end of the 20th century. As the 21st century dawns, automated anesthetics become increasingly prevalent. The closed-loop anesthesia delivery system (CLADS) relies on a completed or “closed” feedback loop. Briefly, an automated device (for example, a ventilator) must be trained to a goal (for example, end-tidal carbon dioxide (CO _2_) level) and govern an input that affects that goal (for example, minute ventilation). The causal interdependence of these factors is the “closed loop”. The common cruise control found in most automobiles is a simple closed-loop system receiving speed as its input and adjusting engine power to achieve a driver-set speed target. As when a car is being driven with the cruise control disengaged, the intraoperative warrant of an anesthetic provider is to make decisions based on data (for example, end-tidal CO _2_) and implement changes (for example, alter minute ventilation via the ventilator). In the US, no truly automated CLADS is approved for commercial clinical use. Still, anesthetic workstations, infusion pumps, and monitors have progressed as increasingly intricate machines that ultimately leave the feedback loop “open” for the anesthesia provider to close themselves. Outside the US, automated CLADSs are employed in research and increasingly in clinical practice ^2^. These systems were first developed separately for specific parameters (for example, processed electroencephalogram (EEG) monitoring, hemodynamic goals, and fluid resuscitation) and have been assessed in specific clinical scenarios, including in patient populations with relatively high comorbidity (cardiac surgery ^3^ and transcatheter aortic valve implantation ^4^). In broader populations, the safety of these systems has been reviewed extensively ^5, 6^. Automated anesthetic systems incorporating independent closed-loops for hypnosis, analgesia, and fluid management are undergoing feasibility studies ^7^. In one multicenter randomized controlled trial of parallel CLADSs using propofol and fentanyl, targeted to the proprietary bispectral index (BIS) and heart rate, the CLADS maintained significantly tighter control than manual operation over BIS ( *P* <0.0001) and heart rate within 25% of baseline ( *P* <0.0031) ^8^. Inter-center variation among these parameters was minimal with automation ( *P* = 0.94) and significant with manual control ( *P* <0.001) ^8^. CLADS controlled total intravenous anesthetic (TIVA) infusions have also been shown to more tightly regulate depth of anesthesia, shorten recovery time, and reduce sedative agent consumption when compared with standard practice in a meta-analysis ^9^. Neurocognitive recovery, a measure of clinical importance beyond the perioperative period, might also be improved under automated TIVA administration ^10^. Pediatric applications are also developing ^11^. The February 2020 issue of *Anesthesiology* featured an editorial“Robots will Perform Anesthesia in the Near Future” ^12^. In some markets, automatic administration of volatile anesthetics via closed-loop titration is commercially available. We expect automation to increase rapidly, becoming the norm in the operating room in the next 10 years.

## Monitoring inside the operating rooms: advances in non-invasive monitoring

Closed-loop automation requires a reliable source of data upon which to make “decisions”. For example, programming a hypothetical capnography-linked ventilator as noted above is a straightforward task governed by well-characterized physiologic principles of CO _2_ production and minute ventilation. These relationships degrade significantly in the presence of data artifact: intraoperative events (for example, pulmonary embolism or myocardial infarction), endotracheal tube malposition, patient temperature, and occluded or disconnected circuit tubing all introduce variations to the capnogram signal and require intervention outside modulation of minute ventilation. Toward this end, the reliable acquisition of high-resolution, reproducible, and timely intraoperative data is foundational to any attempt at automation.

With innovation, monitoring has become less invasive. Cardiac output monitoring, which first required invasive catheters and the use of thermodilution, has been commercially available via analysis of the peripheral arterial pressure waveform for some years. More recently, truly non-invasive assessment of cardiac output became available by using a blood pressure cuff applied to the finger, such as in the CNAP system (CNSystems Medizintechnik GmbH, Graz, Austria) or the ClearSight system (Edwards Lifesciences, Irvine, CA, USA). Cerebral pulse oximetry has similarly unlocked valuable data with the potential for meaningful clinical impact, including brain autoregulation assessment ^13, 14^.

New monitoring technology is not the only means of advanced non-invasive data-gathering. Clinical information sometimes can be derived from established monitors via further in-depth analysis of the data already provided. As with the measurement of cardiac output via pulse-wave contour analysis of the radial arterial line or the treasure trove of data that can be extracted from the electrocardiogram (merely a plot of voltage varying with time), data derived from existing monitors of pulse oximetry, continuous end-tidal CO _2_, arterial pressure hold potentially valuable information to derive hemodynamic variables ^15^. The analgesia nociception index, surgical pleth index, and nociception level index (NoL) are examples ^16, 17^. Edry *et al*. demonstrated a proportional reflection of incisional pain using the NoL, a “nonlinear combination of heart rate, heart rate variability, photoplethysmograph wave amplitude, skin conductance, skin conductance fluctuations, and their time derivatives” ^17^. Because processed metrics like this naturally carry limitations ^18^ that limit their clinical uses (one may expect NoL scores in healthy patients to differ from those with chronic pain), understanding these limitations is prerequisite to their judicious use in closed-loop systems (for example, NoL-guided analgesic administration).

## Monitoring beyond the operating room: telemedicine and wearable health-care technologies

The pursuit of new and more valuable patient data extends beyond the intraoperative setting. Consumer technology companies have poured resources into the developing consumer health-care market. The Consumer Electronics Show (CES), the preeminent trade show in the US for all things tech, now sees regular entries from consumer health-care technology seeking clinical application. Augmented reality technology from the video-gaming industry, for example, has made its way into the intensive care unit (ICU) to quantify patient mobility ^19^. Wireless technologies with low latency and ever-improving stability, developed for the consumer, may someday untangle anesthesiology workstations ^20^.

Telemedicine, a natural by-product of advanced videoconference products in the consumer space, and its application to the perioperative surgical home model of care constitute perhaps the clearest current example of consumer technology revolutionizing anesthesia practice. Small high-resolution cameras, microphones, and broadband data connections necessary for telemedicine have their origins in commerce and espionage. Their use in preoperative examinations, remote ICU care, intraoperative monitoring, and postoperative assessments is reviewed in detail elsewhere ^21^. Data security in consumer health-care technology remains a challenge ^22^, but potential financial savings and sustained patient satisfaction continue to drive the expansion of telemedicine.

## Clinical decision support and anesthesia information management systems

Documentation is a necessary component of the anesthesia provider’s work. Previously, information like vital signs, fluid status, and degree of sedation flowed from the patient to the provider by way of direct observation or through monitors or the anesthesia workstation. Documentation was a one-way act of scribing data for potential review at a later time. Now, the electronic medical record and anesthesia information management systems (AIMS) act as hubs for information gathered by the provider, monitors, and anesthesia workstation. With the anesthetic record becoming a comprehensive repository of real-time patient information, the possibility of clinical decision support (CDS) systems became reality. An extensive review and future perspective on CDS and AIMS have been undertaken elsewhere ^23, 24^. Notably, CDS differs from closed-loop or automated systems in that the CDS provides notification or evidence of best practice in a variety of clinical situations but is not capable of intervention. The anesthesia provider therefore remains indispensable as the source of clinical judgment and intervention.

The fundamental challenges of artificial intelligence (AI) and behavioral science remain as obstacles to an effective CDS implementation. First, an algorithm’s output quality is dramatically altered by the quality of its input (the “garbage in, garbage out” conundrum). Quality patient data and the ability to screen for artifact are vital properties of an effective CDS. Second, CDSs provide various notifications and warnings that rely on the attention of a human provider. Thus, a CDS leaves the feedback loop of clinical care “open”. With alarm fatigue and documentation requirements mounting, there is a sizeable challenge in creating a useful and accurate CDS without it becoming burdensome in practice. Third, an effective CDS requires the processing of gargantuan mounds of clinical data in near real-time. The practicality of housing this processing power locally on an anesthesia workstation remains to be seen, but we suspect that a central processing model similar to that used by web service providers will shift this demand to low-latency networking, enabling greater portability and lower cost of the peri-procedural equipment. This will place demands on the networking and processing capacity already implemented in most practice environments. We expect the advent of consumer-grade low-latency networking (for example, 5G cellular network) to lessen this barrier significantly.

Even as connective and data processing continue to intensify, practices in security and property rights over health-care data will continue to evolve. The human data we create and gather during the 21st century will be unfathomable in scale, scope, and impact. Finding meaning in the data remains a fundamental challenge of our technological age.

## The innovation landscape in anesthesiology technology

The triple aim of health care was launched in 2008 ^25^ and has since guided many efforts in health-care development. While chasing improved outcomes at a lower cost for more patients, technology enjoys several advantages over the traditional pipeline of medical innovation.

Bottom-up innovation and so-called “solution shopping” are common: technology often makes a task or measurement possible before its clinical use, or even its clinical need, is clear. Famously, smart watches and fitness trackers introduced essentially continuous activity and heart rate monitoring, earning applause from society at large and market success. Less developed health-care systems may embrace this technology before advanced systems with pre-existing viable alternatives, leading to a “leapfrog” effect wherein nascent technology proves its validity in developing systems before adoption in advanced health-care settings. Today, clinicians in advanced health-care delivery systems remain faced with uncertain accuracy and reliability of consumer-grade medical information and have yet to codify its use in clinical care.

The fields of psychology, economics, and behavioral science will guide the implementation of the ever-increasing quantities of available patient information. Accounting for human factors and social engineering preserves the anesthesia provider’s most valuable resources: time and attention ^24^. Automated systems must be paired with an understanding of human operator behavior if they are truly going to improve care. Even if implemented with the strongest multi-disciplinary evidence supporting a technology’s utility, use will vary as the discrepancy between evidence and practice continues.

Development costs and regulation persist as necessary barriers to innovation. Development costs for novel technology continue to climb in step with the intricacy of products proposed and must be counteracted in the price of implementation. Regulation continues to function as a basic quality-control measure at the societal level. Markets employ varying standards for the evidence behind new medical technology, but new regulatory pathways aimed at bridging the gap between medical and consumer technology may smooth this process. The US Food and Drug Administration has acknowledged the value of technology transfer between medical and consumer realms, most recently by presenting “Demystifying Regulation” at the CES in January 2020 in an attempt to aid tech startups navigating a complex regulatory framework.

Although some impedance to innovation can be found in prudent quality assurance, philosophical opposition to change is a human habit borne out over centuries with real impact. Technological advance in anesthesiology is an uneasy topic for many practitioners. With innovations like those we have discussed, practitioners can perceive a threat to their purpose and professional identity, perhaps even the reasons for which they dedicated their careers to the service of humanity. Institutions with a vested interest in the status quo (manufacturers, training institutions, and providers) often perceive a threat to power, influence, or prosperity in the face of sweeping change. In his last book, *Innovation and its Enemies*, the late director of the Harvard Kennedy School’s Science Technology and Globalization Project, Calestous Juma, details examples of this preservation instinct combatting waves of technology that upended certain sectors of society. Coffee was banned centuries ago as a substance that encouraged communal gathering and the exchange of ideas. Margarine, a threat to butter sales, was legally required to be dyed a painfully bright shade of pink. Resistance to innovation sometimes leverages coercive force (for example, law) to preserve a way of life, work, or thought. Still, today the café is ubiquitous and selling yellow margarine is not a crime. Anesthesiology’s experience with automation is already complex and under way. We suggest that embracing this wave and responsibly ushering it forward constitute the best way to avoid our own pink margarine legacy.

## Conclusions

As William Gibson famously began to note in the mid 1990’s ^26^, “The future is already here – It’s just not very evenly distributed”. The delivery of anesthesia, its preoperative assessments, and postoperative care vary by health system, resource setting, and society. However, the trends toward automation, non-invasive monitoring, remote monitoring and management, and CDS enabled by AI and improved information technology infrastructure are clear in our field. Each health system, in its setting, will continue to pursue improved outcomes for more patients while expending fewer resources in accordance with the triple aim. Inter-system variation will lead to leapfrog innovation where a set of advances more quickly enacted in one setting will provide the experience used to justify their implementation elsewhere.

Specifically, the CLADS is a mature technology that provides tight control of measurable variables during an anesthetic, but further study is necessary to elucidate clinical relevance (neurocognitive dysfunction aside). Monitoring has become increasingly non-invasive and processing-dependent as we extract novel metrics from proprietary combinations of existing metrics. Telemedicine has the potential to revolutionize the perioperative surgical home model of care and serves as a vanguard for the adoption of consumer-grade technology (telecommunication or otherwise) by medical fields.

Along with technological innovation, social engineering and the constructs of efficient business will help increase quality and value in anesthesia care. Increasingly intricate synthesis of the incredible quantity and breadth of health system and patient metrics will inform this process. Behavioral science and economics will additionally guide the implementation of CDS systems, underpinned by the technologies noted above, with the aims of mitigating provider fatigue and minimizing errors.

Innovation in anesthesiology continues to be driven by the triple aim of health care for the benefit of patients and society. Our approach to innovation as providers and innovators will determine our standing after these developments change our field. We believe that parts of the trends discussed above are inevitable results of economic and social forces acting upon the medical field. We also believe that an informed and alert profession can shape the coming age and guide its members to meaningful and impactful practice. The future is here, and our engagement with innovation will determine our share of its prosperity.
